# Supplementary material for: Impact of host species on assembly, composition, and functional profiles of phycosphere microbiomes
Source: mSystems. 2024 Jul 31;9(8):e00583-24. doi: 10.1128/msystems.00583-24 (PMC11334532; doi:10.1128/msystems.00583-24)
Supplement: Supplemental figures — Fig. S1 to S5. [file msystems.00583-24-s0001.pdf]

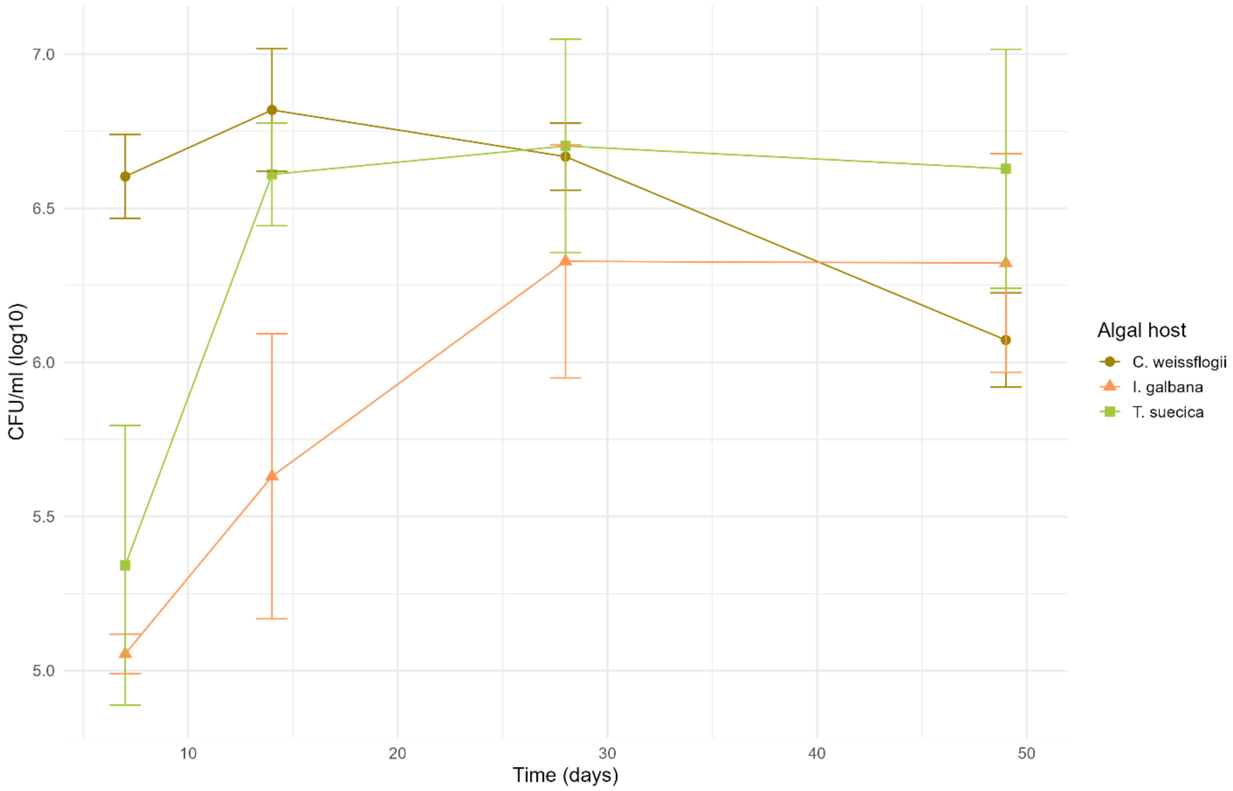

Figure S1. Culturable bacterial counts from microbiomes associated with algal hosts *C. weissflogii* (olive-brown circles), *I. galbana* (orange triangles), and *T. suecica* (green squares) from day 7 to 49. Error bars indicate standard deviations (n = 4).

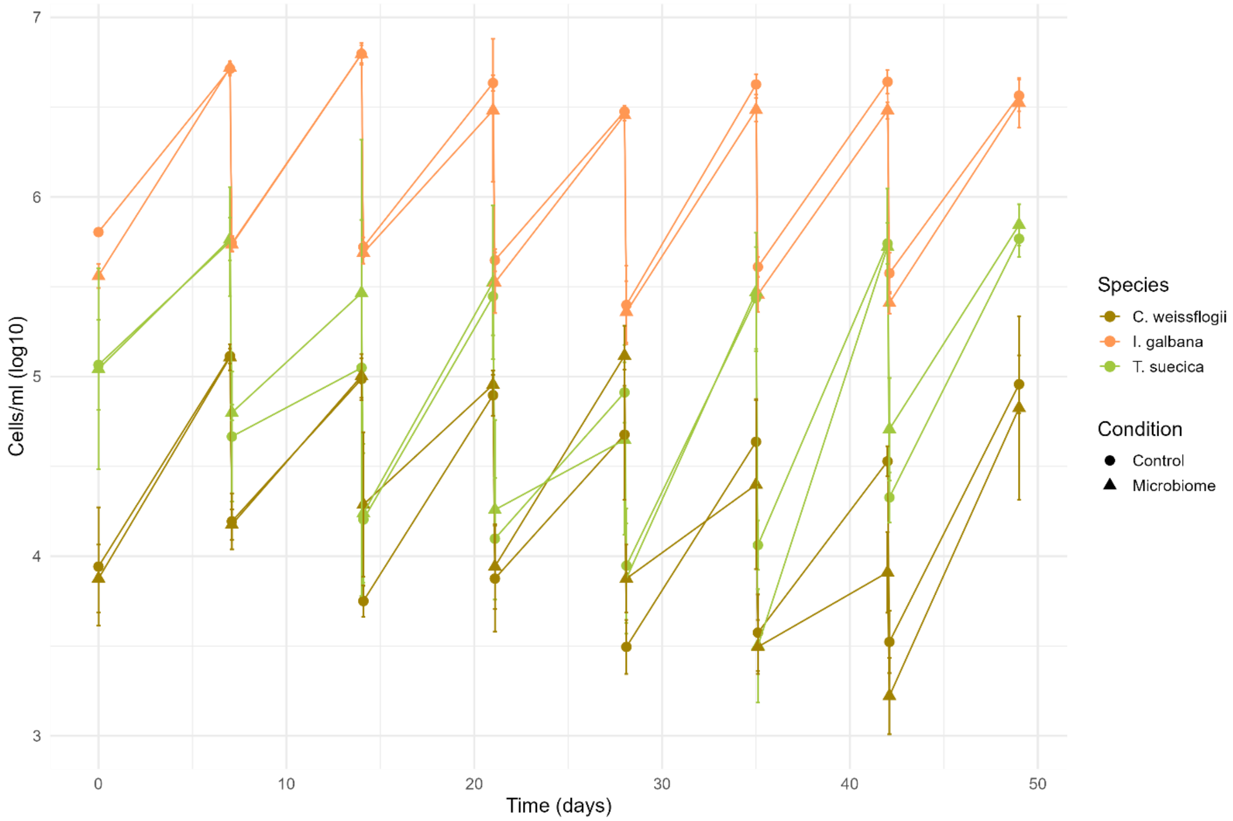

Figure S2. Algal cell counts across the 49-day experiment including 10-fold dilution once per week. Cell concentrations as determined by flow cytometry for *I. galbana* (orange) and *T. suecica* (green) and microscopy counts for *C. weissflogii* (olive-brown). The xenic condition with microbiome (triangles) or axenic control condition (circles) status of the given cultures is indicated by shapes of points. Error bars indicate standard deviations (n = 4).

A.

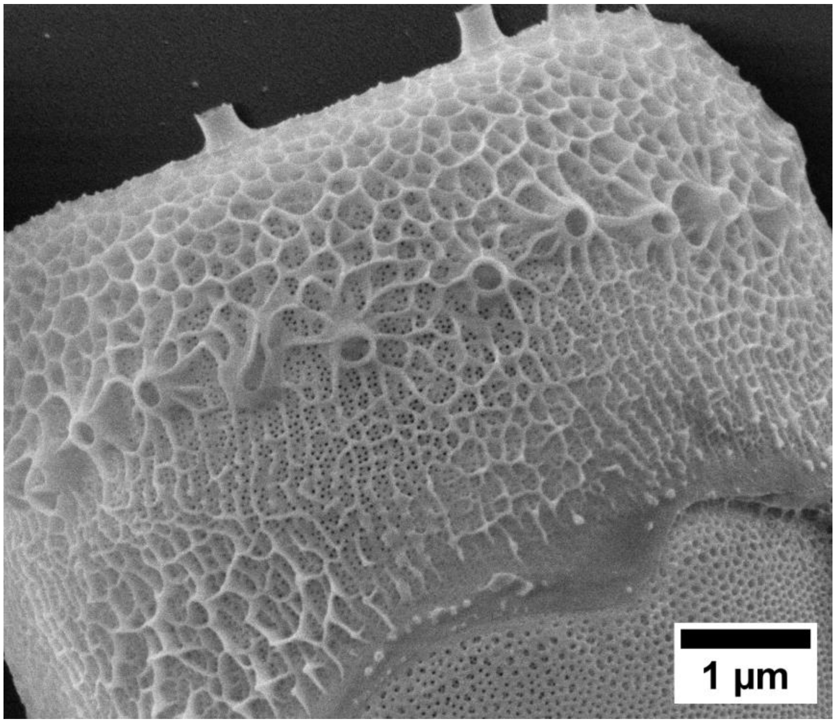

B.

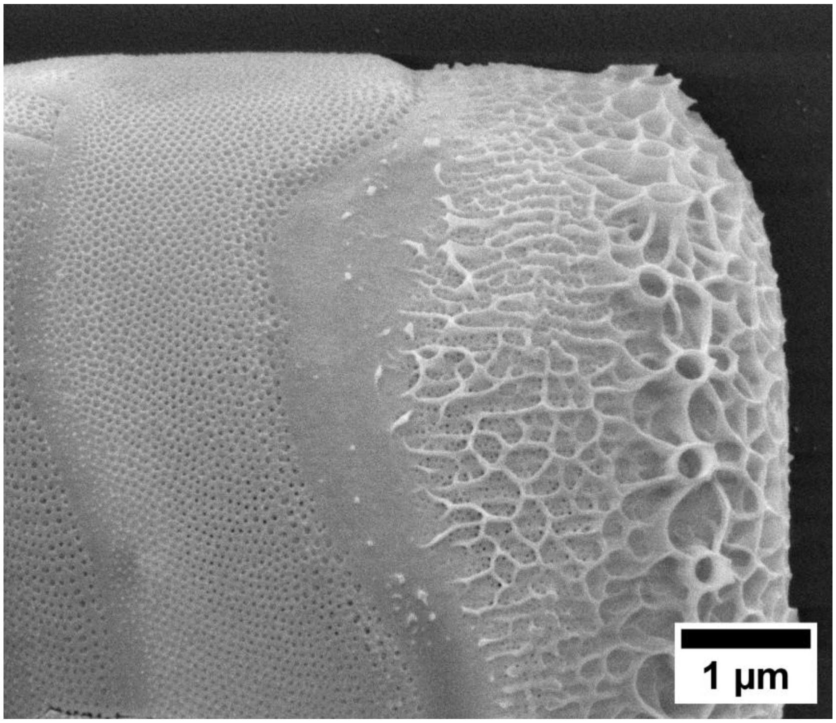

Figure S3. SEM imaging of axenic *C. weissflogii* cells.

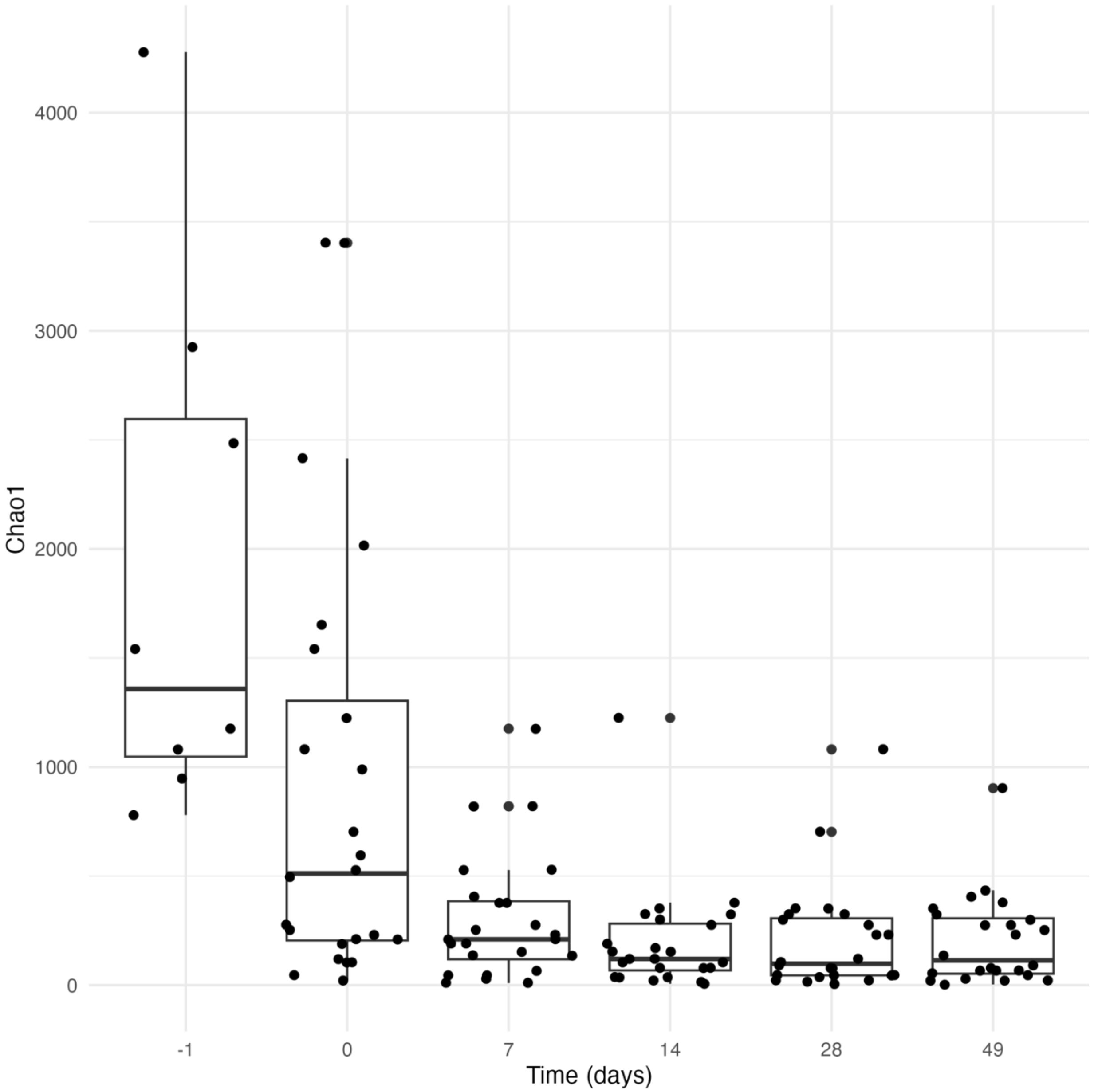

Figure S4. Richness (Chao1) of microbiomes sampled over time associated with the three host microalgae *I. galbana, T. suecica,* and *C. weissflogii.* Time -1 corresponds to the seawater and microbiome inoculum samples.

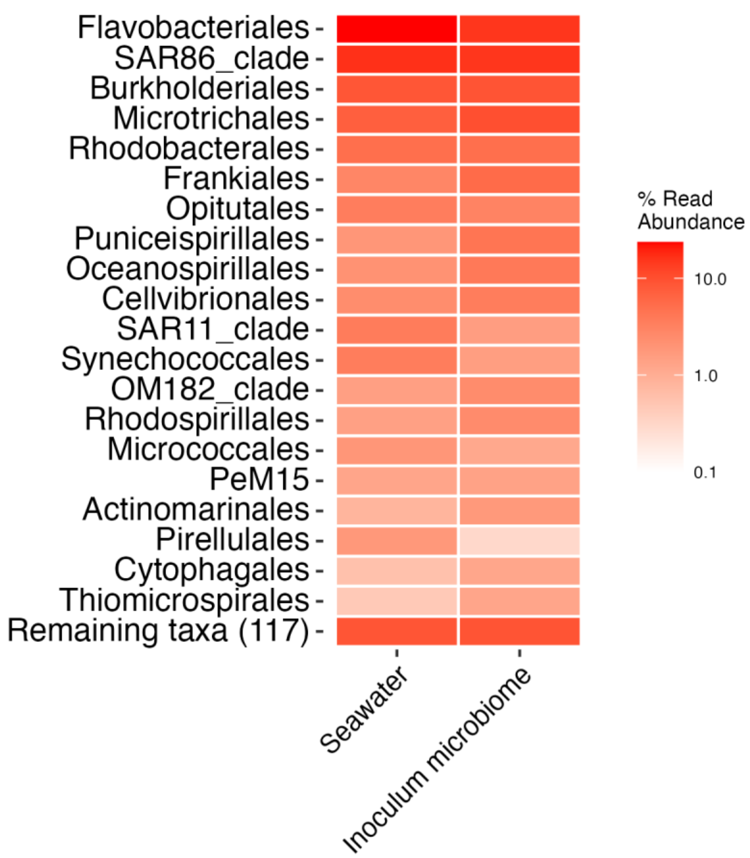

Figure S5. Heatmap relative abundances of top 20 taxonomic orders present in the seawater and inoculum (after filtration at 3 µm) microbiomes introduced to the three host microalgae.
