# Supplementary material for: Impact of host species on assembly, composition, and functional profiles of phycosphere microbiomes
Source: mSystems. 2024 Jul 31;9(8):e00583-24. doi: 10.1128/msystems.00583-24 (PMC11334532; doi:10.1128/msystems.00583-24)
Supplement: Supplemental tables — Tables S1 to S3. [file msystems.00583-24-s0002.docx]

**Table S1.** Overview of samples that underwent 35 PCR cycles to obtain enough yield for 16S rRNA amplicon sequencing. Cw = C. weissflogii, Ig = I. galbana, Ts = T. suecica, and ctrl = negative sequencing control. C or M corresponds to control (axenic) or microbiome inoculum, numbers represent replicate numbers. T0-T49 represents day of the experiment, and A or FL correspond to the attached or free-living fraction of the microbiomes.

| **Sample** |
| --- |
| CwC1_T0_FL |
| CwC3_T0_FL |
| IgM3_T7_FL |
| IgC1_T14_FL |
| IgC4_T14_FL |
| TsC2_T14_FL |
| CwC3_T28_FL |
| IgC3_T28_FL |
| IgC4_T28_FL |
| TsM3_T28_FL |
| CwC2_T49_FL |
| IgM2_T49_A |
| IgC1_T49_FL |
| IgC3_T49_FL |
| IgC4_T49_FL |
| ctrl5 |
| ctrl6 |
| ctrl7 |
| ctrl8 |

**Table S2.** Shannon diversity and Chao1 richness indices of microalgal microbiomes originating from natural seawater as assessed by 16S rRNA gene amplicon sequencing data. Fraction corresponds to either attached (A, captured on 5 µm filters) or free-living (FL, captured on 0.22 µm filters) fractions of the microbiomes.

| **Algal host species** | **Time of sampling (days)** | **Fraction** | **Shannon index** | **Chao1 richness** |
| --- | --- | --- | --- | --- |
| *Conticribra weissflogii* | 0 | A | 3,13 | 387,75 |
| *Conticribra weissflogii* | 0 | FL | 3,40 | 658,75 |
| *Conticribra weissflogii* | 7 | A | 3,44 | 526 |
| *Conticribra weissflogii* | 7 | FL | 3,17 | 449,75 |
| *Conticribra weissflogii* | 14 | A | 2,91 | 193,25 |
| *Conticribra weissflogii* | 14 | FL | 3,36 | 531,50 |
| *Conticribra weissflogii* | 28 | A | 2,94 | 212,50 |
| *Conticribra weissflogii* | 28 | FL | 3,44 | 591,50 |
| *Conticribra weissflogii* | 49 | A | 3,28 | 449 |
| *Conticribra weissflogii* | 49 | FL | 3,22 | 334 |
| *Isochrysis galbana* | 0 | A | 3,11 | 461 |
| *Isochrysis galbana* | 0 | FL | 3,74 | 1227,75 |
| *Isochrysis galbana* | 7 | A | 1,73 | 23,25 |
| *Isochrysis galbana* | 7 | FL | 3,25 | 468 |
| *Isochrysis galbana* | 14 | A | 1,64 | 19,50 |
| *Isochrysis galbana* | 14 | FL | 2,53 | 95,25 |
| *Isochrysis galbana* | 28 | A | 1,75 | 30 |
| *Isochrysis galbana* | 28 | FL | 2,79 | 190,75 |
| *Isochrysis galbana* | 49 | A | 2,42 | 127,50 |
| *Isochrysis galbana* | 49 | FL | 2,61 | 151,50 |
| *Tetraselmis suecica* | 0 | A | 2,87 | 908,50 |
| *Tetraselmis suecica* | 0 | FL | 3,99 | 1809,75 |
| *Tetraselmis suecica* | 7 | A | 2,77 | 152 |
| *Tetraselmis suecica* | 7 | FL | 2,93 | 186,75 |
| *Tetraselmis suecica* | 14 | A | 2,38 | 67,50 |
| *Tetraselmis suecica* | 14 | FL | 3,07 | 263,25 |
| *Tetraselmis suecica* | 28 | A | 2,38 | 93,75 |
| *Tetraselmis suecica* | 28 | FL | 2,42 | 112,75 |
| *Tetraselmis suecica* | 49 | A | 2,43 | 100,50 |
| *Tetraselmis suecica* | 49 | FL | 1,86 | 45,25 |
| Inoculum microbiome | NA | NA | 3,97 | 1536,75 |
| Seawater | NA | NA | 4,09 | 2266,25 |

**Table S3.** Overview of metagenomic sequencing raw reads, host reads, and number of contigs and N50s of assemblies of microalgal microbiome samples at day 49 of the experiment. Sample names represent microalgal host (Cw = C. weissflogii, Ig = I. galbana, and Ts = T. suecica), M for microbiome, and numbers indicate replicate number.

| **Sample** | **Raw reads** | **Host reads (%)** | **# contigs** | **N50** |
| --- | --- | --- | --- | --- |
| CwM1 | 67.9 M | 17.4 | 20,578 | 21,083 |
| CwM2 | 55.0 M | 37.7 | 15,691 | 28,787 |
| CwM3 | 60.4 M | 16.8 | 24,778 | 30,403 |
| CwM4 | 53.6 M | 9.7 | 24,513 | 16,575 |
| IgM1 | 53.4 M | 61.7 | 7,318 | 61,052 |
| IgM2 | 52.6 M | 81.5 | 2,131 | 197,413 |
| IgM3 | 56.8 M | 88.9 | 5,952 | 54,381 |
| IgM4 | 42.4 M | 82.5 | 2,279 | 145,657 |
| TsM1 | 50.7 M | 39.3 | 6,553 | 47,511 |
| TsM2 | 53.4 M | 63.4 | 6,801 | 41,112 |
| TsM3 | 54.0 M | 84.7 | 5,446 | 94,500 |
| TsM4 | 85.5 M | 80.1 | 5,380 | 28,477 |
